# Policy Considerations for National Virtual Hospitals: Global Evidence and the Seha Virtual Hospital Model

**DOI:** 10.2196/89276

**Published:** 2026-06-22

**Authors:** Sohaib Shujaat, Hawazin Almutairi, Saleh Hamad Alhuraibi, Hongyang Ma

**Affiliations:** 1 King Abdullah International Medical Research Center, Department of Maxillofacial Surgery and Diagnostic Sciences, College of Dentistry, King Saud Bin Abdulaziz University for Health Sciences, Ministry of National Guard Health Affairs, Mail Code: 3183; P.O. Box 3660, Riyadh, 11481, Saudi Arabia; 2 King Abdullah International Medical Research Center, College of Dentistry, King Saud Bin Abdulaziz University for Health Sciences, Ministry of National Guard Health Affairs, Riyadh, Saudi Arabia; 3 Department of Oral Implantology, The Second Affiliated Hospital of Harbin Medical University, Harbin, China

**Keywords:** virtual hospitals, telehealth policy, digital health systems, health systems innovation, hospital-at-home, virtual wards, health equity

## Abstract

Health systems worldwide face growing pressure from population aging, multimorbidity, and rising emergency admissions, prompting reconsideration of traditional inpatient care models. In response, digitally enabled models such as tele–intensive care unit (tele-ICU) programs, hospital-at-home services, virtual wards, and other remote specialist pathways have expanded, particularly after the COVID-19 pandemic accelerated telemedicine adoption and cross-site virtual staffing. However, nationally coordinated, multispecialty virtual hospitals remain uncommon worldwide, and robust evidence on their system-level effects is still limited. As a result, policy discussions about national virtual hospitals must often draw on evidence from related virtual-care models rather than from mature national implementations. This viewpoint synthesizes representative international evidence from tele-ICU systems, hospital-at-home programs, virtual wards, telestroke networks, and other condition-specific virtual-care pathways, and examines Saudi Arabia’s Seha Virtual Hospital (SVH) as a national case study to identify policy lessons relevant to the design, governance, and evaluation of national virtual hospitals. Across settings, these models suggest that remote and digitally supported care can achieve outcomes comparable to in-person hospital care when patient selection is appropriate, escalation and transfer pathways are explicit, monitoring intensity matches clinical risk, and multidisciplinary teams are integrated into local workflows. Tele-ICU programs have reported reductions in intensive care mortality and length of stay under well-structured organizational models, while hospital-at-home and virtual-ward programs have shown comparable safety, reduced hospital usage, and improved patient experience among selected patient groups. Telestroke networks likewise demonstrate outcomes comparable to specialist in-person care in acute stroke pathways. Nevertheless, the evidence base remains heterogeneous and strongly context-dependent. Much of the literature is short-term, with limited consistent evidence on long-term outcomes, caregiver burden, cost-effectiveness, workforce implications, and digital equity. SVH illustrates the emerging implementation of a centralized national virtual hospital model. Launched in 2022 under Saudi Arabia’s Vision 2030 Health Sector Transformation Program, SVH operates as a national telehealth hub embedded within the country’s broader digital-health ecosystem and links hospitals across the Kingdom to specialized clinical expertise. Its service portfolio includes urgent and critical care consultations, specialized virtual clinics, multidisciplinary case discussions, and supportive diagnostic services. Early reports indicate rapid operational expansion, broad institutional participation, and national-scale feasibility. However, independent comparative evidence evaluating SVH’s effects on mortality, readmissions, length of stay, cost-effectiveness, equity, and workforce sustainability remains limited. National virtual hospitals should therefore be understood as evidence-generating health-system innovations rather than fully validated care models. Sustainable scale-up requires embedding rigorous prospective evaluation within implementation, aligning financing mechanisms with substitution of inpatient care, establishing clear governance and regulatory frameworks, and addressing digital inclusion and workforce sustainability. These considerations can help guide policymakers and health-system leaders in the accountable, equitable, and evidence-informed development of national virtual hospital programs.

## Introduction

Health systems worldwide face mounting pressure from aging populations, rising multimorbidity, and increasing emergency admissions, prompting reconsideration of traditional inpatient care models [1-3]. Although these pressures are most pronounced in high-income countries, low- and middle-income countries are undergoing parallel epidemiological transitions, with noncommunicable diseases and injuries now accounting for most global mortality and disability and generating sustained hospital demand [4-6]. These trends are driving governments to pursue alternative models of hospital-level care [7]. Hospital-at-home and virtual-ward models have expanded to relieve bed constraints while maintaining clinical outcomes and patient experience [8,9].

The COVID-19 pandemic accelerated this shift through regulatory reform, telemedicine expansion, and normalization of remote clinical decision-making and cross-site virtual staffing [8-11], establishing technical and organizational foundations for more integrated remote hospital care [11]. Within this evolving ecosystem, several virtual-care models have emerged [12]. Tele–intensive care unit (tele-ICU) programs provide centralized specialist oversight of geographically distributed intensive care units (ICUs) through real-time monitoring and structured escalation protocols [13,14]. Virtual wards deliver time-limited hospital-level care remotely to selected patients, typically to support admission avoidance or early discharge [9,15]. Virtual hospitals extend beyond single pathways by integrating tele-ICU, virtual wards, and specialty teleconsultation within shared digital infrastructure, multidisciplinary workforce models, and governance frameworks [9,16].

Evidence from tele-ICU programs demonstrates reductions in ICU mortality and length of stay when effectively integrated into local workflows [13,14,17]. Similarly, hospital-at-home and virtual-ward models show comparable safety to inpatient care, reduced length of stay, and in some contexts lower readmission rates among carefully selected patients [15,18,19]. However, translating this evidence to national, cross-specialty virtual hospitals raises unresolved questions regarding scalability, equity, workforce sustainability, governance, and accountability [15,17]. Differential uptake of telehealth across socioeconomic groups further highlights risks of digital inequity if access barriers are not addressed [20,21]. Although most virtual-hospital implementations remain subnational or specialty-specific, nationally coordinated, cross-specialty models are emerging but are not yet established worldwide [22-24].

This viewpoint examines national virtual hospitals as policy innovations operating in an evidence-generating phase rather than mature digital solutions. Saudi Arabia’s Seha Virtual Hospital (SVH) [25] serves as a national-scale case study. Three key messages guide this analysis: (1) evidence from tele-ICU, hospital-at-home, virtual wards, and condition-specific virtual care provides important but incomplete foundations for national virtual hospitals; (2) national deployment introduces risks related to outcome variability, equity, workforce sustainability, governance, and accountability; and (3) sustainable scale-up requires explicit policy, implementation, and evaluation frameworks. The intended audience includes policymakers, health-system leaders, clinicians, and researchers engaged in digital health transformation.

## Global Evidence Review: System-Level Impact and Evidence Gaps

### Overview

This section synthesizes representative international evaluations on tele-ICU, hospital-at-home, virtual wards, telestroke, and virtual-hospital initiatives. Table 1 summarizes examples of nationally coordinated, multispecialty virtual hospital models. Fully national virtual hospitals remain uncommon internationally, with only a small number of countries, such as Saudi Arabia, Finland, and Jordan, implementing centralized virtual hospital structures [25-27]. In contrast, most jurisdictions rely on national virtual wards, hospital-at-home programs, regional virtual-hospital networks, national telehealth platforms, or institution-led virtual-care systems rather than centralized national virtual hospitals. These broader models are summarized in Multimedia Appendix 1.

**Table 1.** National multispecialty virtual hospital models.

Country	System or entity	Brief description	References
Saudi Arabia	Seha Virtual Hospital	Ministry of Health–led national virtual hospital delivering specialist teleconsultations across public hospitals. The system provides multispecialty and subspecialty services through a centralized digital platform as part of Saudi Vision 2030 health-system transformation.	[25]
Finland	Virtual Hospital 2.0/Health Village (Terveyskylä)	Nationally coordinated digital hospital platform developed by all Finnish university hospitals, delivering condition-specific virtual care pathways, outpatient services, and self-management programs integrated into the public health system. While not delivering inpatient-equivalent care, it functions as a nationally governed virtual hospital platform rather than a generic telehealth service.	[26]
Jordan	Jordan Digital Health Center (virtual hospital)	National virtual hospital network connecting peripheral hospitals to a central command center, providing services such as tele–intensive care unit, teledialysis, and teleradiology, with planned national expansion.	[27]

### Tele-ICU and Remote Critical-Care Networks

Tele-ICU programs represent one of the earliest and most established forms of virtual hospital care [28,29]. Although most tele-ICU systems operate at the institutional or regional rather than national scale, they provide important operational foundations for contemporary virtual-hospital models [30]. Prior studies have reported measurable improvements when tele-ICU services were implemented within structured organizational models. For instance, in a pre-post study of 118,990 patients across 56 ICUs, tele-ICU adoption was associated with approximately 20% reductions in ICU mortality and 15% reductions in hospital mortality, alongside shorter ICU stays [14]. A separate retrospective analysis of 16,091 patients in progressive-care units reported lower unit mortality (0.7% vs 1%), lower hospital mortality (4.4% vs 5.2%), and shorter unit stays (2.6 vs 3.2 d) in telemedicine-supported units, although direct costs were modestly higher [31]. However, larger effectiveness studies evaluating tele-ICU adoption across diverse hospital settings demonstrate more modest and heterogeneous effects. Specifically, in a multicenter study including more than 500 US hospitals, mortality reductions were observed overall but were statistically significant in only a minority of adopting sites [17]. Similarly, a national analysis of 66,522 mechanically ventilated patients found no significant association between tele-ICU availability and in-hospital mortality or duration of ventilation [32]. These findings indicate that tele-ICU systems can produce meaningful outcome improvements under well-defined organizational conditions, but benefits are not uniformly reproducible at scale.

### Hospital-at-Home and Acute Care at Home

Hospital-at-home represents a core component of many contemporary virtual-hospital models [33]. In one trial of acutely ill adults eligible for inpatient admission, substitutive hospital-level care delivered at home was associated with 38% lower direct acute-care costs and reduced 30-day readmissions (23% vs 7%) without increased adverse events [34]. A subsequent pilot trial reported more modest cost reductions (5%) but similarly lower 30-day readmissions (11% vs 36%) while maintaining safety and patient experience [35]. The degree of virtualization within hospital-at-home models also varies. In a randomized clinical trial comparing remote physician visits with traditional in-home visits, remote care was noninferior for adverse events and patient experience, although approximately 20% of patients required occasional in-person physician support [36], suggesting fully virtual oversight may be feasible for many but not all patients.

Across broader evidence syntheses, hospital-at-home models generally demonstrate mortality outcomes comparable to inpatient care across diverse acute conditions, with frequent reductions in length of stay and variable but often favorable cost profiles [37-39]. A review of admission-avoidance hospital-at-home in older adults found little to no difference in six-month mortality, similar readmission rates, and a probable reduction in transitions to residential care [39]. However, caregiver burden and longer-term system effects remain insufficiently characterized. Performance and safety depend heavily on local infrastructure, including trained community nursing teams, workforce capacity, and rapid escalation pathways [40]. Overall, the aforementioned evidence suggests that hospital-at-home can deliver noninferior clinical outcomes, reduced readmissions, and potential cost savings for carefully selected patients, while emphasizing the importance of patient selection and system integration for safe scale-up.

### Virtual Wards and Community-Based Virtual Care

Frail older adults are a major target of virtual-ward programs because of high hospital usage and transitional-care needs [15,41]. Virtual wards extend the hospital-at-home concept by providing time-limited, multidisciplinary oversight for conditions such as frailty, heart failure, and chronic obstructive pulmonary disease [15]. A community-based virtual-ward model in Ireland for older adults with complex needs reported a reduction in median hospital bed-days from 23 to 0 over 12 months, alongside fewer emergency department visits and unplanned admissions, and high patient satisfaction [42]. In England, rapid national implementation of frailty-focused virtual wards has occurred, but comparative outcome data remain limited, and effectiveness appears strongly influenced by local configuration and staffing models [7]. Simulation modeling further suggests that admission thresholds, monitoring intensity, and workforce capacity substantially affect usage and hospital pressure [43].

During the COVID-19 pandemic, multiple virtual-ward and home-monitoring models were deployed at scale. Among these, the national COVID Oximetry @home and COVID Virtual Ward programs in England monitored over 19,000 patients across 26 sites, with per-patient running costs of approximately £528-£599 and mortality below 1% [44]. In Sydney, Australia, a community virtual-care program enrolled 93.6% of locally diagnosed COVID-19 patients, with low escalation rates (ambulance 3%, emergency department 2.5%, and hospital admission 1.9%) and no reported deaths [45]. These experiences demonstrate the feasibility of virtual-ward deployment during periods of extreme health-system pressure, while highlighting the importance of strong clinical governance, integration with primary and community care services, and adequate workforce support [46]. Overall, the aforementioned evidence suggests that virtual-ward models may reduce unplanned hospital usage and facilitate earlier discharge for selected patient groups. However, most evaluations remain uncontrolled, context-specific, and short-term, with limited long-term clinical, economic, and caregiver data [44-46].

### Telestroke and Other Condition-Specific Virtual-Care Models

Among condition-specific virtual-care pathways, telestroke has one of the most robust comparative evidence bases, consistently demonstrating safety and effectiveness comparable to in-person specialist care for acute ischemic stroke [47]. A meta-analysis of 1863 patients treated within the 3-hour therapeutic window found no significant differences between telemedicine-guided and in-person thrombolysis in symptomatic intracerebral hemorrhage, mortality, or 3-month functional independence [48]. In a hub-and-spoke telestroke network (3‐4.5-hour window), rates of symptomatic intracerebral hemorrhage (4.9% vs 6.3%), functional independence (44.1% vs 39.6%), and in-hospital mortality (9.6% vs 8.3%) were comparable to tertiary stroke-center care [49]. A larger meta-analysis including 12,540 patients similarly reported no meaningful differences in 90-day mortality or functional independence between telestroke and in-person management [47].

Beyond stroke, other condition-specific virtual-care models show potential to reduce usage and improve access. Among these, a wraparound virtual-care program for medically complex children reported 35.3% fewer hospitalizations and 43.9% fewer emergency department visits over 12 months in a pre-post evaluation of 75 patients, although the absence of concurrent controls limits causal inference [50]. In addition, a four-year panel analysis of 1.6 million admissions across 63 hospitals showed that telehealth adoption was associated with shorter hospital stays, with greater benefits at longer travel distances (≈11-minute reduction in length of stay per additional minute of travel time) [51]. Across tele-ICU, hospital-at-home, virtual-ward, and telestroke pathways, a consistent pattern emerges where virtual-care models can match in-person outcomes when embedded within structured networks, clear clinical pathways, and appropriate workforce support [13,18,47]. However, effects remain context-dependent, indicating that technology alone does not ensure system-level benefit [17,29].

## System-Level Considerations and Health-System Impact

### Outcomes and Workload Redistribution

The effects of virtual hospitals on outcomes and workload depend on how care delivery is organized across the health system [17]. Reductions in hospital usage reported in frailty-focused virtual wards appear most consistent when services are tightly integrated with primary care, multidisciplinary teams are stable, and escalation pathways are clearly defined [15]. When these structural elements are absent, effects are variable [15,17]. Clinicians often value virtual wards, but report increased cognitive load, coordination complexity, and uncertainty when protocols are poorly defined [52]. Broader digital-health research shows similar dynamics, where electronic health interventions may reduce in-person visits while increasing workload through additional messaging, documentation, and coordination [53]. High volumes of secure messaging and video consultations have also been associated with increased burnout among primary-care physicians [54]. These findings suggest that virtual hospitals redistribute rather than simply reduce workload.

Ambient artificial intelligence scribe technologies have been proposed as one potential mitigation strategy [55]. By generating clinical documentation from real-time encounters, early deployments report reductions in note-writing time and after-hours documentation burden [55,56]. However, integration remains evolving, and regulatory issues related to accuracy, privacy, and accountability persist [57]. Such tools may therefore complement virtual-hospital models but cannot substitute for structural workforce design and clear governance. As national virtual-hospital programs expand, formal evaluation should incorporate workload metrics alongside usage and cost outcomes, recognizing that reductions in inpatient bed-days do not necessarily equate to net workforce relief [53,54].

### Equity and Digital Determinants of Health

Digital determinants of health describe how access to digital devices and connectivity, digital literacy, platform usability, trust, and affordability influence who can benefit from digital health interventions [58]. These aforementioned determinants interact with traditional social determinants of health and may either mitigate or exacerbate existing health inequities. From this perspective, virtual hospitals are not neutral technological solutions but system-level care models whose impact depends on how digital resources and capabilities are distributed across populations [58,59]. The digital equity framework suggests that virtual care has the potential to expand access and reduce travel burdens, particularly for rural and underserved communities; however, evidence of sustained equity gains remains limited, and many initiatives continue to underserve individuals experiencing socioeconomic disadvantage [59].

Digitalization may further widen or reduce disparities among people with disabilities, depending on accessibility standards, affordability, and the availability of appropriate technical and social support [60]. These concerns are reflected in real-world usage patterns, as patient-portal adoption is consistently lower among individuals with lower income, older adults, minority populations, and those with limited health literacy [61]. As virtual hospital models often assume reliable connectivity, access to digital devices, and caregiver support, digital determinants of health must be explicitly incorporated into program design and evaluation rather than treated as implicit or automatic benefits [58,60].

### Workforce Well-Being and Organizational Dynamics

Sustainable virtual-hospital models depend heavily on organizational conditions, particularly because virtual care often redistributes rather than removes clinical work [52,53,62]. Burnout among health care professionals is strongly associated with heavy workloads, inadequate staffing, and limited organizational support [62]. In virtual-ward deployments, staff describe tension between enthusiasm for home-based care and anxiety about responsibility for deteriorating patients outside hospital walls [52]. These concerns are especially relevant for national virtual hospitals, where centralized specialist input may increase coordination demands on local teams [52]. Workforce planning should therefore include realistic staffing models, clear role delineation, escalation support, protected time for virtual responsibilities, and structured clinical and emotional support [52,62].

### Governance, Regulation, and Accountability

Governance and regulatory requirements for virtual hospitals are jurisdiction-dependent but share common challenges, including clinician licensure, scope of practice, data protection, cross-border teleconsultation, and clinical liability [63,64]. These factors directly affect safety, scalability, and public trust. In Saudi Arabia, the Personal Data Protection Law and Telehealth Application Guidelines establish requirements for data processing, privacy, documentation, and secure workflows, while professional licensure remains governed by national regulatory authorities [65-67]. International frameworks such as the European Union’s General Data Protection Regulation, the US HIPAA (Health Insurance Portability and Accountability Act), and the World Health Organization (WHO) telemedicine guidance similarly emphasize legal clarity, data protection, and professional accountability in digital care [68-70].

Beyond regulation, accountability requires structured evaluation. WHO and related frameworks recommend staged assessment from pilot to scale-up, addressing effectiveness, equity, unintended harms, and economic impact [71,72]. For national virtual hospitals, this requires prospective and comparative designs rather than reliance on usage or satisfaction metrics alone [71]. Despite growing policy attention, most evaluations remain short-term and usage-focused, with limited evidence on equity, caregiver burden, workforce effects, long-term outcomes, and system-level financing [29,73-78].

### Evidence Gaps

The evidence across tele-ICU, hospital-at-home, virtual-ward, and condition-specific models reveals persistent uncertainties regarding scalability, sustainability, and system-level impact [17,73,78]. First, clinical outcomes are heterogeneous and highly context-dependent [17,29]. While some tele-ICU studies reported mortality benefits, national analyses demonstrated only modest overall mortality reductions with substantial heterogeneity across hospitals after adjustment for case mix and hospital characteristics, suggesting that effects vary by patient selection, program design, workforce capacity, and system readiness, thereby limiting generalizability [17]. Methodological constraints, including selection bias, residual confounding, and short follow-up, further complicate interpretation [29]. Second, evidence in frail older adults, the primary target of virtual wards and hospital-at-home, remains mixed, where multidisciplinary home-based interventions show inconsistent effects on mortality, adverse events, and emergency usage, and transitional-care gains often attenuate over time [73-75]. Third, most evaluations emphasize 30-90–day outcomes, leaving uncertainty regarding long-term disease control, functional trajectories, cost-effectiveness, and unintended consequences such as over-surveillance or centralization of expertise [73-77]. Comparative-effectiveness evidence remains limited by heterogeneous intervention components and inconsistent outcome definitions [29]. Fourth, caregiver and social impacts are rarely measured, as caregiver strain, financial burden, and informal-care capacity are seldom incorporated into evaluation frameworks despite concerns about shifting responsibility to families [77-79]. Taken together, current evidence supports cautious optimism but does not yet establish consistent, long-term, system-level benefit for national virtual hospital deployment [17,73,78].

## Policy Principles for National Virtual Hospitals

### Embed Programs Within National Digital-Health Strategy and Governance

WHO’s Global Strategy on Digital Health emphasizes that digital initiatives should be embedded within broader health-system goals and supported by governance structures that address interoperability, equity, and trust [80]. Telehealth implementation frameworks similarly show that successful programs require attention not only to technology but also to organizational change, financing, user perceptions, and legal context [81]. Recent Australian work further demonstrates how national digital-health strategies can be mapped against value-based health care principles, highlighting priority populations, standardized outcome measurement, and patient-centered metrics as key processes for embedding value into digital care [82]. Another framework (Planning and Evaluating Remote Consultation Services) for remote consultations reinforces the need to integrate patient, clinical, organizational, and system perspectives, and highlights practical ethics, including distributive justice and data governance [83]. For virtual hospitals, this implies that expansion should be grounded in explicit strategy and regulation rather than driven solely by local innovation or vendor offerings [81-83].

### Align Financing and Incentives With Substitution of Care

Financing design is central to the sustainability of national virtual hospitals. During the COVID-19 pandemic, many countries reimbursed telehealth at parity with in-person care and relaxed geographic restrictions, enabling rapid scale-up but raising concerns about long-term sustainability and potential overuse [84]. Evidence from value-based payment models shows that reimbursement structures determine whether telehealth substitutes for high-cost inpatient care or simply generates additional billable encounters [85,86]. National virtual hospital financing must therefore incentivize substitution, such as replacing inpatient bed-days with virtual care, while avoiding duplication and excess usage [86].

Economic sustainability should be embedded in program design and evaluation [87]. Standard health-economic methods, including cost-utility analysis using quality-adjusted life years, incremental cost-effectiveness ratios, and multiyear budget impact modeling, are directly applicable [44,71,72,87,88]. Evidence from hospital-at-home and virtual hospital-in-the-home programs suggests that remote acute care can reduce length of stay and, in selected populations, total costs without compromising outcomes [9,18,19,34-37]. Modeling and budget impact analyses further indicate potential cost savings and quality-adjusted life year gains when substitution effects outweigh additive usage [77,87,89]. Tele-ICU programs have similarly reported cost reductions associated with shorter ICU stays and fewer complications [31,90,91].

### Prioritize Equity and Digital Inclusion in Design and Deployment

A digital-health equity framework argues that digital determinants of health, such as connectivity, device access, digital literacy, and trust, interact with traditional social determinants to shape who benefits from digital care [58]. Telehealth can be described as a “double-edged sword,” capable of reducing or reinforcing inequities depending on implementation [92]. Telehealth in China also highlights practical barriers, including technology costs, variable internet quality, and limited digital skills, particularly in rural areas [93]. Consequently, virtual hospital policies should embed equity by providing device and connectivity support where feasible, ensuring accessible interfaces, maintaining low-tech options such as telephone-based services, and routinely collecting disaggregated equity data [58].

### Make Evaluation a Core Design Feature Rather Than an Afterthought

Digital programs are expected to follow a staged evaluation pathway from feasibility and pilots to pragmatic effectiveness and scale-up [71,72]. The Pan American Health Organization telemedicine framework recommends multidimensional evaluation across clinical effectiveness, patient experience, economics, organizational functioning, and socioethical or legal domains [94]. Evaluation questions for national virtual hospitals should be defined at inception, including impacts on mortality, functional outcomes, readmissions, caregiver burden, equity, and patient experience [71,72,94,95]. Linked data systems and independent academic collaboration are essential to ensure transparent reporting, including null or negative findings [71,72]. These principles highlight that national virtual hospitals are system-level interventions whose value depends on governance, financing, equity, and workforce sustainability rather than technology alone, and provide a framework for interpreting the SVH experience [80,81,83].

## SVH as a National Case Study

### Overview

SVH was launched in February 2022 under Saudi Arabia’s Vision 2030 Health Sector Transformation Program as a national-scale virtual hospital initiative. SVH operates as a centralized telehealth hub connecting hospitals with specialized clinical services across the Kingdom [25]. It is embedded within a broader national digital-health ecosystem developed by the Ministry of Health. This ecosystem includes the Seha application for remote consultations and the Sehhaty platform for access to personal health records and digital health services [96,97]. Care coordination is further supported by national infrastructure, including the 937 unified health hotline for teleconsultation and triage, the Mawid platform for appointment scheduling and referrals, and complementary systems such as Health Electronic Surveillance Network for disease surveillance, Tawakkalna for health-status verification, and Wasfaty for electronic prescribing and medication dispensing [98].

### Operational Model and Clinical Workflow

**Figure 1.**
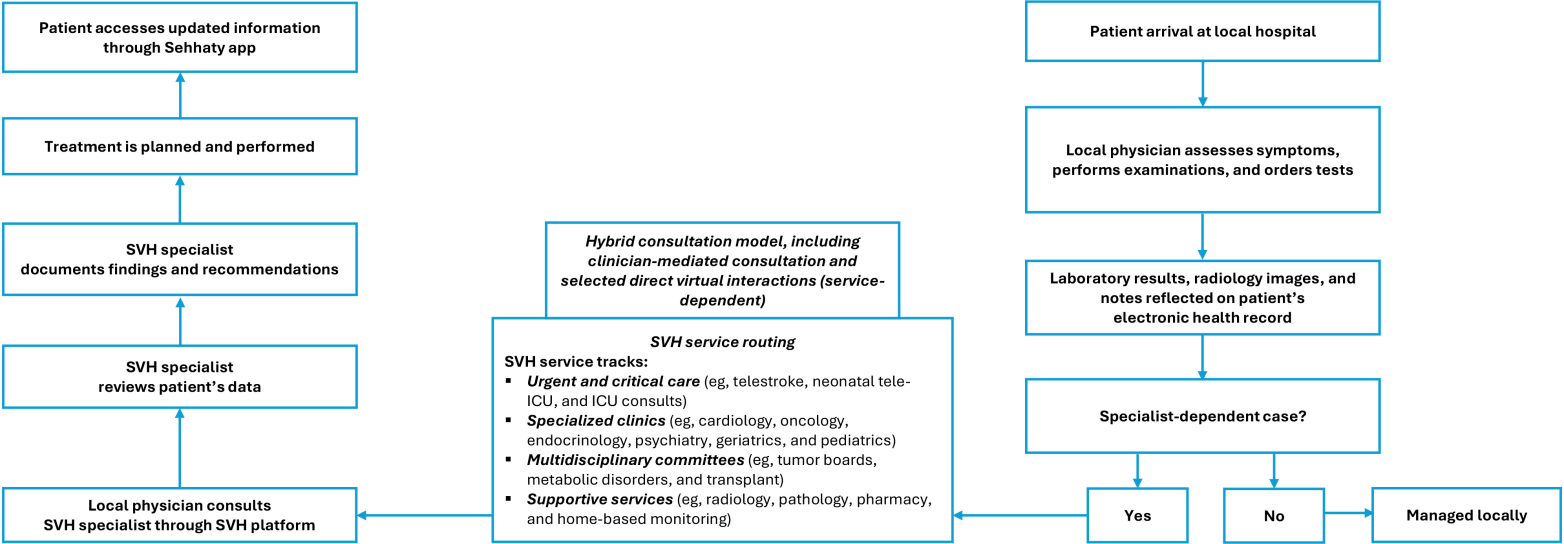
SVH operational workflow. ICU: intensive care unit; SVH: Seha Virtual Hospital.

Unlike many localized or specialty-specific models, SVH operates as a national, multispecialty virtual hospital within Saudi Arabia’s digital-health infrastructure, enabling examination of feasibility, operational scalability, and national-level integration across multiple clinical domains [99,100]. Operationally, SVH functions as a fully digital tertiary-care network connecting local hospitals, patients, and centralized specialist expertise through clinician-mediated and direct specialist-patient teleconsultations [96,99,100]. In many inpatient and high-acuity services, care begins when a patient presents to a local hospital, where the attending physician conducts a clinical assessment, orders diagnostic investigations, and documents findings [99]. Laboratory results, radiological images, and clinical notes are integrated within electronic health record systems and related digital-health platforms supporting the SVH network [97,99]. When specialist input is required, the local physician initiates a consultation through SVH, and the SVH specialist remotely reviews clinical data, provides recommendations, and documents them in the shared electronic health record [99]. Treatment is subsequently implemented locally, while patients access results, prescriptions, and care plans via Sehhaty [97]. In addition, SVH delivers real-time virtual specialist–patient consultations, including urgent or critical care pathways (eg, telestroke), virtual specialty clinics (eg, cardiology and psychiatry), and live remote consultations supported by secure video links at local sites [25,100] (Figure 1).

### Scope of Services and Organizational Structure

As a key Vision 2030 initiative, SVH currently supports more than 170 hospitals, delivers services across 29 core specialties and 73 subspecialties, employs over 150 physicians, and has an annual capacity exceeding 480,000 patients [25,100]. The range of services spans clinical functions typically concentrated in tertiary referral settings and is delivered through coordinated virtual care streams [99,100]. Services are organized into four virtual tracks: urgent and critical care consultations, specialized clinics, multidisciplinary committees, and supportive medical services, ensuring timely access to expertise, improved efficiency of care, and knowledge transfer at scale [25,100]. Urgent and critical care services include time-sensitive presentations such as acute stroke, seizures, neonatal telecritical care, and intensive-care consultations [25,101,102]. Specialized clinics manage chronic and complex conditions across cardiology, endocrinology and diabetes, oncology, nephrology, hematology, geriatrics, psychiatry, rehabilitation medicine, and metabolic and genetic disorders [25,99]. Multidisciplinary committees support complex case-based decision-making, for example, through virtual heart or diabetes teams [25]. Supportive services include diagnostic reporting (radiology and pathology), pharmaceutical services, and home-based care pathways such as hospital-at-home models and remote physiological monitoring [25,99]. Collectively, these services allow SVH to function as a national tertiary hub for specialist consultation, diagnostic support, and integrated clinical management across geographically distributed hospitals [25,99,100].

### Emerging Evidence and Evaluation Gaps

Given SVH’s recent national rollout [25], independent evidence on long-term clinical, economic, equity, and workforce outcomes remains limited. Current SVH-related literature is largely descriptive, documenting growth in case volumes, service expansion, and national adoption, and positioning SVH as a central component of Saudi Arabia’s telemedicine infrastructure [99]. While these findings demonstrate feasibility and operational scalability, they do not establish whether SVH improves clinical outcomes or overall system performance. The absence of external comparators further limits assessment of effectiveness and cost-effectiveness, and it remains unclear whether observed efficiencies reflect true substitution of inpatient care or expansion of service activity.

Early reports suggest pathway-specific benefits, including improved workflow in neonatal telecritical care and successful telestroke deployment during Hajj [101,102]. Health care workers report generally positive perceptions, although challenges related to connectivity, workload, and training persist [103]. Earlier Saudi tele-ICU studies show improved short-term outcomes [104], and national surveys indicate broad patient acceptance alongside usability concerns among older or digitally less confident populations [105-107]. However, these findings remain insufficient to establish a consistent system-level impact. It is therefore unclear whether SVH reduces avoidable hospital usage, redistributes workload, or introduces additional coordination demands. Similarly, the extent to which virtual consultations alter referral thresholds, escalation patterns, and interhospital transfers has not been systematically evaluated.

A central unresolved policy question is whether national virtual hospitals function primarily as substitutive models that reduce inpatient demand or as additive layers that increase overall system activity [77,86]. This distinction has direct implications for cost-effectiveness, workforce burden, and long-term sustainability [77]. Without robust comparative evaluation, apparent efficiency gains may reflect improved access or expanded usage rather than genuine system-level improvement [108]. Addressing these uncertainties requires prospective, comparative, and system-level evaluation designs, including standardized outcome measures, appropriate control groups, longitudinal follow-up, and explicit assessment of substitution effects, patient trajectories, equity, and workforce impact [71,72,94].

### Conceptual Implications

SVH aligns with global virtual-hospital trends through centralized specialist support, integration within a national digital-health strategy, and deployment in high-demand contexts such as mass gatherings [22,80,101,102]. However, its scale and scope suggest that national virtual hospitals should not be understood merely as telemedicine platforms, but as system-level reorganizations of care delivery [22,70,80]. This configuration may expand access to advanced care and reduce unnecessary transfers, particularly in underserved regions, but it also introduces new requirements for governance, coordination, and medico-legal accountability across distributed teams [24,63,64,69]. Conceptually, national virtual hospitals may be conceptualized as involving three interacting system transformations: (1) centralization of specialist expertise, (2) decentralization of care delivery, and (3) digital integration across care pathways. This triad distinguishes virtual hospitals from earlier telehealth models, which typically operate at the level of individual services rather than coordinated systems [11,22,70]. Unlike traditional hospitals defined by physical infrastructure, national virtual hospitals are distributed, network-based systems in which clinical expertise is decoupled from location but remains embedded within local care pathways [22,23]. Responsibility for clinical decision-making may therefore be distributed between virtual specialists and on-site providers, necessitating clearly defined professional roles, escalation pathways, documentation standards, and accountability mechanisms [63,64,66,69]. Conventional metrics such as bed occupancy, admission rates, or service volumes may fail to capture key system effects, including care redistribution, avoided transfers, changes in clinical decision-making, and coordination across networks [22,77]. Evaluation frameworks should therefore prioritize system-level indicators such as substitution of care, coordination efficiency, network-wide outcomes, equity of access, workforce impact, and cost-effectiveness [71,72,83,94]. Aligning evaluation with the distributed nature of virtual hospital care is essential for accurate assessment and accountable policy decision-making [71,83,94].

## Interpreting Current Evidence and the Role of SVH

The international literature supports a cautious interpretation of national virtual hospitals. Outcomes vary according to patient selection, workforce capacity, escalation protocols, workflow integration, and digital maturity [41,52,77]. In this context, Saudi Arabia’s SVH contributes primarily evidence of national feasibility and integration [99,100]. As a cross-specialty program embedded within a national digital ecosystem, SVH illustrates how specialist expertise can be distributed across geographically dispersed hospitals and linked to national platforms for records, scheduling, triage, and prescribing [25,97-99]. However, the current evidence base remains early-stage [99]. Hence, SVH should not yet be treated as a settled exemplar of effectiveness. The central policy question is not whether SVH can operate at scale, but whether its expansion translates into demonstrable system-level value when evaluated using rigorous prospective frameworks rather than descriptive growth metrics alone [109,110].

## Policy Implications: Financing, Governance, Equity, and Workforce

The system-level nature of national virtual hospitals creates four immediate policy priorities. First, financing must be aligned with substitution rather than addition [86]. Without clear definitions of virtual activity and reimbursement structures that reward avoided admissions, reduced bed-days, or fewer transfers, programs risk increasing total encounters without commensurate system benefit [108]. Embedding transparent economic evaluation alongside implementation is therefore essential [71]. Second, governance and regulation must be operationalized [69]. National programs require clear definitions of clinical responsibility, licensing, scope of practice, documentation standards, data access, privacy safeguards, and liability across virtual and in-person teams, particularly in clinician-mediated models [66]. Third, equity should be treated as a design requirement rather than an assumed benefit [58]. National deployment offers an opportunity to measure and address digital and social inequities systematically, but gains require stratified reporting and targeted inclusion strategies [20,58]. Fourth, workforce sustainability must be explicitly monitored [62]. Evaluation should include staffing requirements, role evolution, burnout metrics, and the net impact on local teams [52]. Complementary innovations such as ambient documentation tools may reduce administrative burden, but their integration, safety, and governance implications require careful evaluation rather than an assumption of benefit [97].

## From Service Delivery to a Learning Health System

A central implication of the SVH case is that national virtual hospitals are well-positioned to operate as learning health systems. The most credible route to resolving these uncertainties is to embed prospective evaluation into program operations, including pragmatic phased rollouts of new service lines, linked-data comparative analyses, and mixed-methods implementation research that explains why effects vary across sites and populations [71,72,83]. This agenda also supports the case for clinician-researcher roles and structured partnerships with academic institutions (and, where appropriate, industry), enabling transparent reporting of benefits, null findings, and unintended consequences [111]. Such an approach would move national virtual hospitals beyond descriptive growth metrics toward policy-relevant evidence on outcomes, equity, workforce impact, and value [71,72]. Despite these opportunities, several limitations warrant consideration.

This viewpoint synthesizes representative international evidence to inform policy interpretation rather than to provide exhaustive comparative analysis. Accordingly, conclusions reflect patterns across selected studies and may not capture the full range of findings across all contexts. Interpretation of the SVH case is further constrained by the current availability of publicly reported data, which remains largely descriptive and short-term. As a policy-oriented analysis, this paper emphasizes system-level implications and governance considerations rather than direct comparative evaluation. Robust prospective and comparative assessments will be required to determine long-term effectiveness, equity impact, economic value, and workforce sustainability on a national scale.

## Conclusions

National virtual hospitals are emerging as policy responses to rising hospital demand, workforce constraints, geographic inequities, and the need for flexible specialist care. Evidence from tele-ICU programs, hospital-at-home services, virtual wards, telestroke networks, and other virtual-care models suggests that digitally enabled care can achieve outcomes comparable to conventional hospital care when supported by appropriate patient selection, structured monitoring, clear escalation pathways, and effective integration into clinical workflows. However, the evidence base remains heterogeneous and context-dependent, with important gaps regarding long-term outcomes, cost-effectiveness, caregiver burden, workforce sustainability, digital equity, and whether virtual care substitutes for or adds to existing health care usage.

Saudi Arabia’s SVH demonstrates the feasibility of a nationally coordinated, multispecialty virtual hospital embedded within a broader digital-health ecosystem, illustrating how centralized expertise can support geographically distributed care and improve access to specialist services. Nevertheless, SVH remains in an evidence-generating phase, as current evidence is largely descriptive and focused on operational expansion rather than robust comparative evaluation of clinical outcomes, equity, workforce impact, and economic value. National virtual hospitals should therefore be viewed not simply as technological innovations, but as complex health-system interventions requiring clear governance, sustainable financing, regulatory accountability, and explicit attention to digital inclusion and workforce capacity. Embedding prospective, comparative, and system-level evaluation frameworks within implementation will be essential to determine whether these models deliver measurable improvements in outcomes, efficiency, equity, and overall health-system performance.

## Supplementary material

10.2196/89276 Multimedia Appendix 1 International virtual-care models beyond national multispecialty virtual hospitals.

## Abbreviations

HIPAA: Health Insurance Portability and Accountability Act
ICU: intensive care unit
SVH: Seha Virtual Hospital
tele-ICU: tele–intensive care unit
WHO: World Health Organization
