# Supplementary material for: Policy Considerations for National Virtual Hospitals: Global Evidence and the Seha Virtual Hospital Model
Source: J Med Internet Res. 2026 Jun 22;28:e89276. doi: 10.2196/89276 (PMC13286081; doi:10.2196/89276)
Supplement: Multimedia Appendix 1 — International virtual-care models beyond national multispecialty virtual hospitals. [file jmir-v28-e89276-s001.docx]

**Identification of International Virtual Care Models**

Hospital/ models included in Table 1 and Appendix 1 were identified through targeted narrative searches of PubMed and Google Scholar up to January 2025, using combinations of keywords such as “virtual hospital,” “virtual ward,” “hospital-at-home,” “tele–ICU,” “telehealth,” “telemedicine,” “remote patient monitoring,” and “national digital health.” Grey literature was also purposively reviewed, including official government health portals, national health-system websites, and policy documents.

Inclusion was guided by relevance to hospital-level or specialist virtual care delivery at national, regional, or institutional scale. The table is intended to provide illustrative international examples that support the policy discussion rather than a comprehensive catalogue of global virtual-care initiatives. Consistent with the Viewpoint nature of the article, formal systematic-review methods and risk-of-bias assessment were not applied.

**Table S1.** International virtual-care models beyond national multi-specialty virtual hospitals.

| **National virtual wards / national hospital-at-home programmes** | | | | |
| --- | --- | --- | --- | --- |
| National virtual ward | United Kingdom | National Health Service (NHS) Virtual Wards Programme | Nationally coordinated NHS programme delivering hospital-level care to patients in their own homes through digitally enabled monitoring and remote clinical oversight. The initiative supports acute and frailty-related pathways and is integrated across acute, community, and primary care services. | [1] |
| National virtual ward | Ireland | Health Service Executive (HSE) National Virtual Ward Programme | Nationally led HSE programme delivering hospital-level care to patients in their own homes through digitally enabled monitoring and multidisciplinary clinical oversight. The programme was initially implemented at selected hospital sites and is being expanded as part of a broader national hospital-at-home strategy. | [2] |
| National Hospital at Home policy | U.S. | Centers for Medicare & Medicaid Services (CMS) Acute Hospital Care at Home (AHCaH) | Federal Medicare waiver programme that allows eligible hospitals to deliver acute inpatient-level care in patients’ homes under defined safety, quality, and reporting requirements, while maintaining Medicare reimbursement equivalent to in-hospital care. | [3] |
| **Regional / state-level virtual hospitals and virtual-care networks** | | | | |
| Regional virtual hospital | Australia | Royal Prince Alfred (RPA) Virtual / Sydney Local Health District Virtual Hospital | Local Health District–level virtual hospital providing virtual care, including virtual emergency department, COVID and chronic-disease monitoring and post-acute follow-up across Sydney Local Health District. | [4] |
| Regional / state-supported virtual wards and hospital-at-home programmes | Australia | State-supported virtual ward and hospital-at-home initiatives (e.g., Royal Melbourne Hospital, Austin Health) | State-supported, institution-led virtual ward and hospital-at-home services delivering acute and post-acute care through remote monitoring and multidisciplinary clinical oversight, including programs for heart failure, post-cardiac care, and outreach to regional and rural communities. | [5] |
| Provincial virtual care | Canada | Real-Time Virtual Support (RTVS) & telehealth network | Provincially coordinated virtual-care pathways offering real-time specialist support (emergency, maternity, pediatrics, mental health, etc.) for rural and Indigenous communities; not branded as a single “virtual hospital” but functioning as a provincial virtual-specialist layer. | [6] |
| Provincial virtual care | Canada | Provincial tele-ICU and hospital-at-home initiatives | Provincially supported tele-ICU and virtual critical-care services for remote and smaller hospitals, alongside expanding hospital-at-home and virtual-ward pilot programmes implemented across multiple regional health systems in Ontario. These initiatives are coordinated at the provincial level but delivered through local health organizations. | [7] |
| State virtual hospital | Germany | Virtuelles Krankenhaus North Rhine-Westphalia (NRW) | State-funded virtual hospital in North Rhine-Westphalia providing tele-ICU, infectious-disease and other specialist teleconsultations across hospitals in NRW. | [8] |
| Regional hospital-at-home | Spain | Hospital-at-Home units (e.g. Hospital Clínic Barcelona) | Hospital-led Hospital-at-Home units delivering substitutive inpatient care at home for acute medical and post-surgical patients; widely implemented regionally but not under a national VH brand. | [9, 10] |
| Regional virtual wards | New Zealand | Hospital-in-the-Home and regional virtual-care pilots | Regional health services under Te Whatu Ora (Health New Zealand) operate hospital-in-the-home and virtual-care pilots, including remote patient monitoring and home-based acute and chronic care. These services are regionally implemented, variable in scope, and not part of a nationally standardized virtual-ward programme, which remains under development and subject to funding constraints. | [11] |
| **National telehealth / virtual-care platforms (not branded as “virtual hospital”)** | | | | |
| National telemedicine platform | India | eSanjeevani – National Telemedicine Service | Government-run national telemedicine platform offering doctor-to-doctor and doctor-to-patient consultations, fully integrated into India’s digital health infrastructure. | [12] |
| National internet-hospital ecosystem | China | Internet Hospitals & national telemedicine network | Nationwide ecosystem of online hospitals enabled by “Internet+ Healthcare” policies, delivering virtual consultations, follow-up care, and e-prescribing through distributed providers rather than a single central entity. | [13] |
| National telehealth | Brazil | Telessaúde Brasil Redes / PROADI-SUS telehealth | A national telehealth network linking primary-care teams with specialists through teleconsultation and tele-education within the public health system. | [14,15,16] |
| National digital-health platform | Singapore | National digital health infrastructure (Smart Nation / HealthHub / National Electronic Health Record) | National digital-health infrastructure supporting teleconsultations, remote monitoring, e-prescribing, and integrated care delivery across the public health system. This ecosystem enables system-wide virtual care but is not organized as a single centralized national virtual hospital. | [17,18,19] |
| National telehealth ecosystem | Denmark | National telehealth integration (municipal and regional services) | Nationally coordinated telehealth ecosystem integrating telemedicine and remote patient monitoring across municipal, regional, and national levels of the Danish health system. Large-scale programs (e.g. telemonitoring for chronic obstructive pulmonary disease and heart failure) are supported by shared national digital-health infrastructure and interoperability frameworks. These services enable system-wide virtual care delivery but are not organized or branded as a centralized virtual hospital. | [20,21] |
| National telehealth ecosystem | Estonia | National digital health and telehealth services | Nationally integrated digital health ecosystem enabling virtual consultations, e-prescribing, e-referrals, and remote follow-up through a nationwide electronic health record and e-government infrastructure. Teleconsultations and digital care pathways are embedded across primary and specialist care, functioning as a national virtual-care backbone rather than a centralized virtual hospital. | [22] |
| National virtual-care ecosystem (emerging) | United Arab Emirates | Ministry of Health and Prevention (MoHAP) virtual-care services | Federally coordinated virtual-care services delivered through Ministry of Health and Prevention digital platforms, including virtual clinics and telemedicine consultations providing multi-specialty care across the public health system. These services represent an emerging national virtual-care ecosystem rather than a centralized national virtual hospital. | [23] |
| National telehealth strategy | Portugal | National TeleHealth Centre / National Telehealth Plan | National telehealth strategy coordinated through the National TeleHealth Centre and integrated into the Serviço Nacional de Saúde (SNS). Telehealth services are implemented across SNS providers within a nationally coordinated digital-health framework, supporting system-wide integration of care rather than a single centralized virtual hospital. | [24] |
| National telehealth strategy | Chile | National Telehealth Program | National telehealth program integrating remote consultations into the public health network; used here as an example of national virtual-care infrastructure. | [25] |
| National tele-consultation | Qatar | Hamad Medical Corporation (HMC) / Primary Health Care Corporation (PHCC) virtual-care services | National teleconsultation and virtual-care services delivered through Qatar’s public healthcare system, including primary care, specialty services, mental health, and chronic disease management via HMC and PHCC digital platforms. These services function as an integrated virtual-care layer within the national health system rather than a centralized national virtual hospital. | [26,27,28] |
| National telehealth / virtual-care platform | United Kingdom | NHS digital and virtual-care services | Nationally coordinated digital and virtual-care infrastructure delivering digital triage, remote consultations, and condition-specific virtual pathways via NHS digital platforms. These services complement the NHS Virtual Wards Programme but do not constitute a centralized or formally designated national virtual hospital. | [29] |
| National telehealth / virtual-care platform | United Kingdom | NHS Scotland – *Near Me* Video Consulting Service | National, government-led video consultation platform implemented across all NHS Scotland Board areas, supporting primary and secondary care. Initially developed for rural access and rapidly scaled during COVID-19, *Near Me* functions as a national virtual-care backbone enabling secure browser-based consultations, rather than a hospital-level virtual care or inpatient substitution model. | [30] |
| **Institutional / single-system virtual hospitals (non-national)** | | | | |
| Institutional virtual hospital | U.S. | Mercy Virtual Care Center | “Hospital without beds” providing virtual ICU, hospitalist and chronic-disease management services for multiple Mercy hospitals across several US states. | [31] |
| Institutional hospital-at-home | U.S. | Mayo Clinic – Advanced Care at Home | Multi-site acute hospital-at-home model combining in-home visits with continuous telemonitoring and remote physician oversight. | [32] |
| Institutional virtual hospital | U.S. | Tampa General Virtual Hospital | Health-system virtual hospital / hospital-at-home model delivering hospital-level care at home in Florida. | [33] |
| Institutional hospital-at-home | U.S. | Cleveland Clinic Hospital-at-Home / virtual-care programmes | Large integrated health system with hospital-at-home and extensive virtual-care services across various locations. | [34,35] |
| Institutional virtual hospital | Israel | Sheba Beyond | Institution-led virtual hospital operated by Sheba Medical Center, delivering remote hospitalization, virtual clinics, chronic-care management, and international telemedicine. While it operates at national and international scale, it is governed by the institution rather than by a national health authority. | [36] |
| Institutional / enterprise virtual-care ecosystem (non-national) | United Arab Emirates | PureHealth virtual-care and digital health ecosystem | Enterprise-led virtual-care and digital health ecosystem operated by PureHealth, integrating telemedicine services, healthcare cloud infrastructure, data platforms, and remote-care technologies across a large network of public–private healthcare providers. While operating at national scale and supporting system-wide digital transformation, governance and service delivery are organizational rather than federally administered. | [37] |
| Institutional / enterprise virtual-care system | U.S. | Veterans Health Administration (VHA) Telehealth Services | Federally operated, enterprise-wide virtual-care system delivering tele-primary care, tele-mental health, tele-specialty care, and a national tele-ICU network across VHA medical centers. Although operating at national scale, the program is governed as a single integrated healthcare system and functions as an institutional virtual-care ecosystem rather than a national virtual hospital. | [38] |

**References**

1. NHS England. Virtual wards. <https://www.england.nhs.uk/virtual-wards/> [accessed January 28, 2026].
2. Health Service Executive (Ireland). National virtual ward programme. <https://www.hse.ie/eng/about/who/strategic-programmes-office-overview/national-virtual-ward-programme/> [accessed January 28, 2026].
3. Centers for Medicare & Medicaid Services. Acute Hospital Care at Home data release fact sheet. <https://www.cms.gov/newsroom/fact-sheets/acute-hospital-care-home-data-release-fact-sheet> [accessed January 28, 2026].
4. Hutchings OR, Dearing C, Jagers D, Shaw MJ, Raffan F, Jones A, et al. Virtual health care for community management of patients with COVID-19 in Australia: observational cohort study. J Med Internet Res. 2021;23(3):e21064. PMID:33687341 doi:10.2196/21064
5. Victorian Department of Health (Australia). Virtual hospital pilot. <https://www.health.vic.gov.au/patient-care/virtual-hospital-pilot> [accessed January 28, 2026].
6. Rural Coordination Centre of BC. Rural Telehealth Virtual Services (RTVS). <https://rccbc.ca/initiatives/rtvs/> [accessed January 28, 2026].
7. Ontario Telemedicine Network. Virtual critical care. <https://otn.ca/providers/emergency-services/virtual-critical-care/> [accessed January 28, 2026].
8. Ministerium für Arbeit, Gesundheit und Soziales Nordrhein-Westfalen. Virtuelles Krankenhaus. <https://www.mags.nrw/virtuelles-krankenhaus> [accessed January 28, 2026].
9. González-Colom R, Carot-Sans G, Vela E, Espallargues M, Hernan G, Jiménez FX, et al. Five years of Hospital at Home adoption in Catalonia: impact, challenges, and proposals for quality assurance. BMC Health Serv Res. 2024;24(1):154. PMID:38297234 doi:10.1186/s12913-024-10603-1
10. Hospital Clínic Barcelona. Hospital at Home. <https://www.clinicbarcelona.org/en/service/hospital-at-home> [accessed January 28, 2026].
11. Health Informatics New Zealand. National virtual care programme subject to budget challenges. <https://www.hinz.org.nz/news/677347/National-virtual-care-programme-subject-to-budget-challenges.htm> [accessed January 28, 2026].
12. Sood S, Lal K, Bhatia M, Kapoor G, Singh S, Kaushish RK, et al. Adoption and utilization of India’s eSanjeevani national telemedicine service. Oxf Open Digit Health. 2025;7:oqaf025. PMID:41158553 doi:10.1093/oodh/oqaf025
13. Zhang M, Dai D, Hou S, Liu W, Gao F, Xu D, Hu Y. Thinking on the informatization development of China’s healthcare system in the post–COVID-19 era. Intell Med. 2021;1(1):24-28. PMID:34777904 doi:10.1016/j.imed.2021.03.004
14. Telehealth Brazil Networks Project (LAIS, UFRN). Telehealth Brazil Networks. <https://lais.huol.ufrn.br/en/projetos/telehealth-brazil-networks/> [accessed January 28, 2026].
15. Pereira Silva D, Figueiredo Matos EH, Costa de Paula LG, Santos de Paiva TM, Soares LM, Silva MF, et al. The experience of telehealth in the SUS from the perspective of specialized care projects. *Rev Aracê.* 2025;7(9):1-19. doi:10.56238/arev7n9-050
16. Ministry of Health (Brazil). Telessaúde. <https://www.gov.br/saude/pt-br/composicao/seidigi/sus-digital/telessaude> [accessed January 28, 2026].
17. Smart Nation Singapore. Smart Nation Singapore. <https://www.smartnation.gov.sg/> [accessed January 28, 2026].
18. National University Health System (Singapore). NUHS at Home. <https://www.nuhs.edu.sg/patient-care/nuhs-at-home> [accessed January 28, 2026].
19. Ministry of Health Technologies (Singapore). MIC Home. <https://www.moht.com.sg/our-programmes/integrated-general-hospital/mic-home/> [accessed January 28, 2026].
20. Danish Ministry of Health and the Interior. *A coherent and trustworthy health network for all.* <https://www.ism.dk/Media/637643704565970267/A%20coherent%20and%20trustworthy%20health%20network%20for%20all.pdf> [accessed January 28, 2026].
21. Vestergaard AS, Hansen L, Sørensen SS, Jensen MB, Ehlers LH. Is telehealthcare for heart failure patients cost-effective? An economic evaluation alongside the Danish TeleCare North heart failure trial. BMJ Open. 2020;10(1):e031670. PMID:31992604 doi:10.1136/bmjopen-2019-031670
22. e-Estonia. E-health records. <https://e-estonia.com/solutions/e-health-2/e-health-records/> [accessed January 28, 2026].
23. Ministry of Health and Prevention (UAE). MOHAP launches virtual clinics to further strengthen its telemedicine system. <https://mohap.gov.ae/en/w/mohap-launches-virtual-clinics-to-further-strengthen-its-telemedicine-system> [accessed January 28, 2026].
24. Serviços Partilhados do Ministério da Saúde (Portugal). *PENTS (Plano Estratégico Nacional de Telemedicina e Saúde Digital)* [PDF]. <https://www.spms.min-saude.pt/wp-content/uploads/2019/11/PENTS_Tradu%C3%A7%C3%A3o.pdf> [accessed January 28, 2026].
25. Ministerio de Salud (Chile). *Programa Nacional de Telesalud* [PDF]. <https://portalsaluddigital.minsal.cl/wp-content/uploads/2022/06/Programa-Nacional-de-Telesalud.pdf> [accessed January 28, 2026].
26. Hamad Medical Corporation (Qatar). Virtual consultations. <https://hamad.qa/EN/Hospitals-and-services/HMC-Mental-Health-Service/Our-Services/Pages/Virtual-Consultations.aspx> [accessed January 28, 2026].
27. Primary Health Care Corporation (Qatar). Video consultations. <https://www.phcc.gov.qa/patients-clients/eservice-guestpage/video-consultations> [accessed January 28, 2026].
28. Hamad Medical Corporation (Qatar). Qatar launching new remote healthcare services. <https://hamad.qa/EN/news/2020/March/Pages/Qatar-launching-new-remote-healthcare-services.aspx> [accessed January 28, 2026].
29. NHS Digital. NHS Digital. <https://digital.nhs.uk/> [accessed January 28, 2026].
30. World Health Organization. *Near-me video consultations during the COVID-19 pandemic: Scotland, United Kingdom* [PDF]. <https://www.who.int/docs/librariesprovider2/default-document-library/scotland-united-kingdom-near-me-video-consultations-during-the-covid-19-pandemic-(2021).pdf> [accessed January 28, 2026].
31. Mercy. Virtual care. <https://www.mercy.net/service/virtual-care/> [accessed January 28, 2026].
32. Mayo Clinic. Hospital at Home overview. <https://www.mayoclinic.org/departments-centers/hospital-at-home/sections/overview/ovc-20551797> [accessed January 28, 2026].
33. Tampa General Hospital. Virtual health. <https://www.tgh.org/institutes-and-services/virtual-health> [accessed January 28, 2026].
34. Cleveland Clinic. Connected Care services. <https://my.clevelandclinic.org/departments/connected-care/services> [accessed January 28, 2026].
35. Cleveland Clinic Florida. Hospital Care at Home. <https://my.clevelandclinic.org/florida/departments/medicine/depts/hospital-care-at-home> [accessed January 28, 2026].
36. Zychlinski N, Fluss R, Goldberg Y, Zubi D, Barkai G, Zimlichman E, Segal G. Tele-medicine controlled hospital at home is associated with better outcomes than hospital stay. PLoS One. 2024;19(8):e0309077. PMID:39159148 doi:10.1371/journal.pone.0309077
37. PureHealth. PureHealth. <https://purehealth.ae/> [accessed January 28, 2026].
38. U.S. Department of Veterans Affairs. VA Telehealth Services. <https://telehealth.va.gov/> [accessed January 28, 2026].
